# Evaluating robotic actions: spatiotemporal brain dynamics of performance assessment in robot-assisted laparoscopic training

**DOI:** 10.3389/fnrgo.2025.1535799

**Published:** 2025-02-19

**Authors:** Katharina Lingelbach, Jennifer Rips, Lennart Karstensen, Franziska Mathis-Ullrich, Mathias Vukelić

**Affiliations:** ^1^Applied Neurocognitive Systems, Fraunhofer Institute for Industrial Engineering IAO, Stuttgart, Germany; ^2^Applied Neurocognitive Psychology, Department of Psychology, Carl von Ossietzky University, Oldenburg, Germany; ^3^Department Artificial Intelligence in Biomedical Engineering, Friedrich-Alexander-University, Erlangen, Germany

**Keywords:** robot training, performance monitoring, spatio-temporal clustering, temporal decoding, machine learning, electroencephalography (EEG), passive brain-computer interfaces (BCIs), current source density (CSD)

## Abstract

**Introduction:**

Enhancing medical robot training traditionally relies on explicit feedback from physicians to identify optimal and suboptimal robotic actions during surgery. Passive brain-computer interfaces (BCIs) offer an emerging alternative by enabling implicit brain-based performance evaluations. However, effectively decoding these evaluations of robot performance requires a comprehensive understanding of the spatiotemporal brain dynamics identifying optimal and suboptimal robot actions within realistic settings.

**Methods:**

We conducted an electroencephalographic study with 16 participants who mentally assessed the quality of robotic actions while observing simulated robot-assisted laparoscopic surgery scenarios designed to approximate real-world conditions. We aimed to identify key spatiotemporal dynamics using the surface Laplacian technique and two complementary data-driven methods: a mass-univariate permutation-based clustering and multivariate pattern analysis (MVPA)-based temporal decoding. A second goal was to identify the optimal time interval of evoked brain signatures for single-trial classification.

**Results:**

Our analyses revealed three distinct spatiotemporal brain dynamics differentiating the quality assessment of optimal vs. suboptimal robotic actions during video-based laparoscopic training observations. Specifically, an enhanced left fronto-temporal current source, consistent with P300, LPP, and P600 components, indicated heightened attentional allocation and sustained evaluation processes during suboptimal robot actions. Additionally, amplified current sinks in right frontal and mid-occipito-parietal regions suggested prediction-based processing and conflict detection, consistent with the oERN and interaction-based ERN/N400. Both mass-univariate clustering and MVPA provided convergent evidence supporting these neural distinctions.

**Discussion:**

The identified neural signatures propose that suboptimal robotic actions elicit enhanced, sustained brain dynamics linked to continuous attention allocation, action monitoring, conflict detection, and ongoing evaluative processing. The findings highlight the importance of prioritizing late evaluative brain signatures in BCIs to classify robotic actions reliably. These insights have significant implications for advancing machine-learning-based training paradigms.

## 1 Introduction

Current research is advancing the development and optimization of robotic systems capable of autonomously performing specialized tasks and providing adaptive assistance to support surgeons during various stages of procedures (Moustris et al., 2011; Richter et al., 2019; Thananjeyan et al., 2017). These tasks include camera guidance (Pandya et al., 2014), tissue clamping (Nguyen et al., 2019), tissue manipulation (Scheikl et al., 2024), and surgical knot tying (Osa et al., 2014; Van Den Berg et al., 2010).

Machine learning, particularly reinforcement learning, is well-suited for training robots efficiently, allowing them to learn tasks autonomously (Iturrate et al., 2010; Vukelić et al., 2023). A key challenge, however, lies in providing effective feedback to the reinforcement learning agent. The agent requires frequent and continuous evaluation of its actions via a reward function to distinguish between successful and unsuccessful outcomes. Reinforcement learning is typically trained in simulated environments using this reward function before being adapted to real-world settings for fine-tuning or deployment. The design of the reward function and the real-world fine-tuning both rely on the expertise of physicians. However, obtaining explicit feedback in the form of labels for robot actions from physicians is challenging, as it further burdens their already demanding workload.

Passive brain-computer interfaces (BCIs) offer a promising approach by enabling direct, implicit and continuous feedback loops in human-robot interactions (e.g., Aricò et al., 2018; Protzak et al., 2013), thereby alleviating the burden on physicians (Zander et al., 2017). Brain signals elicited during the observation and mental assessment of robot actions can serve as an evaluation function for reinforcement learning models (Kim et al., 2017; Vukelić et al., 2023).

Previous studies on performance monitoring tasks, including those in BCI applications (Chavarriaga et al., 2010; Iturrate et al., 2015; Ehrlich and Cheng, 2019; Ferrez and Millán, 2005; Ferrez and Millán, 2008; Kreilinger et al., 2012; Spüler and Niethammer, 2015), have shown that observing errors is associated with pronounced event-related potential (ERP) deflections, particularly in the following components (see Somon et al., 2017 for review): Across various tasks, an observation-based error-related negativity (oERN; Somon et al., 2017) has been consistently identified, resembling the ERN observed in self-generated errors (Gehring et al., 1993; also referred to as error negativity (Ne) in early studies; Falkenstein et al., 1991). However, the oERN peaks slightly later, between 250 and 270 ms, in frontocentral regions and is enhanced in response to erroneous actions (Chavarriaga et al., 2010; Ferrez and Millán, 2005; Ferrez and Millán, 2008; Somon et al., 2017; Pavone et al., 2016).

The oERN is sometimes followed by a frontocentral positivity known as error positivity (oPe), which responds to errors depending on contextual factors such as task engagement and error relevance. This component tends to be absent when another observed agent produces the error without relational impact or direct consequence for the observer (Chavarriaga et al., 2010; van Schie et al., 2004; Koban et al., 2010; Padrao et al., 2016). The oPe peaks between 350 and 450 ms and is thought to reflect conscious recognition and high-level evaluation of errors (Ferrez and Millán, 2005; Ferrez and Millán, 2008; Somon et al., 2017; Pavone et al., 2016).

Many of the studies on error monitoring in observed agents and systems (Ferrez and Millán, 2005; Chavarriaga et al., 2010; Padrao et al., 2016; Pavone et al., 2016) have identified a further negative ERP deflection, likely linked to prediction violations and unexpected events. This monitoring-related ERP termed the interaction ERN by Ferrez and Millán (2005), peaks at frontocentral sites between 400 and 550 ms and is proposed to be related to the N400. Initially linked to semantic inconsistencies, the N400 typically peaks around 450 ms post-stimulus at centroparietal sites (Kutas and Hillyard, 1980). However, it has also been observed in non-semantic contexts, such as unexpected outcomes in movement sequences, with a more frontocentral and temporoparietal distribution (Balconi and Vitaloni, 2014).

Building on this foundation, promising results have emerged in training non-medical robots using these error-related ERPs (Iturrate et al., 2010, 2015; Kim et al., 2017, 2020; Luo et al., 2018; Penaloza et al., 2015; Salazar-Gomez et al., 2017; Vukelić et al., 2023). Despite these advances, the application of BCI-based training for medical robots in realistic scenarios remains scarce.

This study investigated evoked spatiotemporal dynamics associated with evaluating optimal and suboptimal robot actions during a robot-assisted laparoscopic simulation using electroencephalography (EEG). Our objectives were twofold: (a) to determine whether the spatiotemporal dynamics evoked by observing optimal and suboptimal robotic actions in near-naturalistic laparoscopic robot training videos resemble commonly reported error-related potentials, using two complementary analytical approaches; and (b) to identify the optimal time interval of these evoked brain signatures for single-trial classification, with potential application for feedback loops in BCI-driven reinforcement learning systems.

## 2 Materials and methods

### 2.1 Participants

Sixteen volunteers (*M*_*age*_ = 24.88 years, *SD* = 4.88, range: 19–38 years, 14 females, two males) with no prior experience in surgical procedures participated in the study. Eligibility criteria included age between 18 and 40 years, right-handedness, absence of diagnosed neurological, physiological, or psychological disorders, no regular use of centrally acting substances, and no head implants or history of brain surgeries. Participants provided written informed consent before participation and received monetary compensation. The study complied with the Declaration of Helsinki and was approved by the University of Tübingen Ethics Committee (ID: 827/2020BO1).

### 2.2 Procedure

At the beginning of the experiment, EEG signals were recorded during a 2-min resting period while participants focused on a fixation cross with their eyes open. Participants subsequently undertook an evaluation task, requiring them to observe laparoscopic video sequences and mentally assess the quality of the robotic action depicted in each sequence.

**Figure 1:**
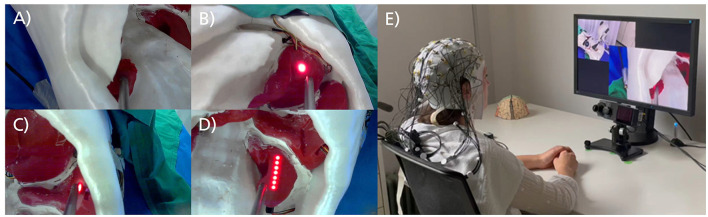
Excerpt from zoomed-in video sequences and laboratory setup. **(A)** Video sequence of the right kidney with a point sensor. The LED light turned off during the video, indicating the robot's action was successful. **(B)** Video sequence of the stomach with a point sensor. The sensor was not touched during the video, and the robot's action failed. **(C)** Video sequence of the spleen with a point sensor. The sensor was touched during the video, but the pressure was insufficient, resulting in a failed action. **(D)** Video sequence of the left kidney with a line sensor. The line sensor was not touched during the video, leading to a failed action. **(E)** Laboratory setup with a participant seated in front of the monitor and eye-tracking system, wearing a 64-channel EEG.

The video sequences illustrated simulated tissue-cutting procedures performed by a robotic arm using a rod instead of a scalpel. These procedures were conducted on a phantom torso model with replicated organs, offering realistic representations of robotic actions in laparoscopic surgery. Light-emitting diodes (LEDs) were used to mark the target organ and tissue for the surgical cut. The target organs included the right kidney, stomach and spleen, each equipped with a single LED point sensor, and the left kidney, which was fitted with a line sensor consisting of a row of seven LEDs (Figures 1A–D). The optimal action required the robot to press the rod with sufficient pressure onto the target organ for the point sensor and to move the rod along the organ's surface for the line sensor. If the robot applied adequate pressure to the marked tissue, the LEDs turned off (Figure 1A). Conversely, if the tissue was missed or the pressure was insufficient, the LEDs remained fully or partially lit. Detailed information about the stimulus material and an illustrative overview video are provided in the Supplementary material. The stimulus database is accessible upon request through the OSF repository at https://osf.io/6ndsv/.

Participants rated each robotic action as good or bad, giving verbal responses during the practice phase to confirm task comprehension, and conducting mental evaluations during the actual experiment. High classification accuracy of robot action assessments was confirmed in a preliminary behavioral study (*N* = 9; see Supplementary material for details).

**Figure 2:**
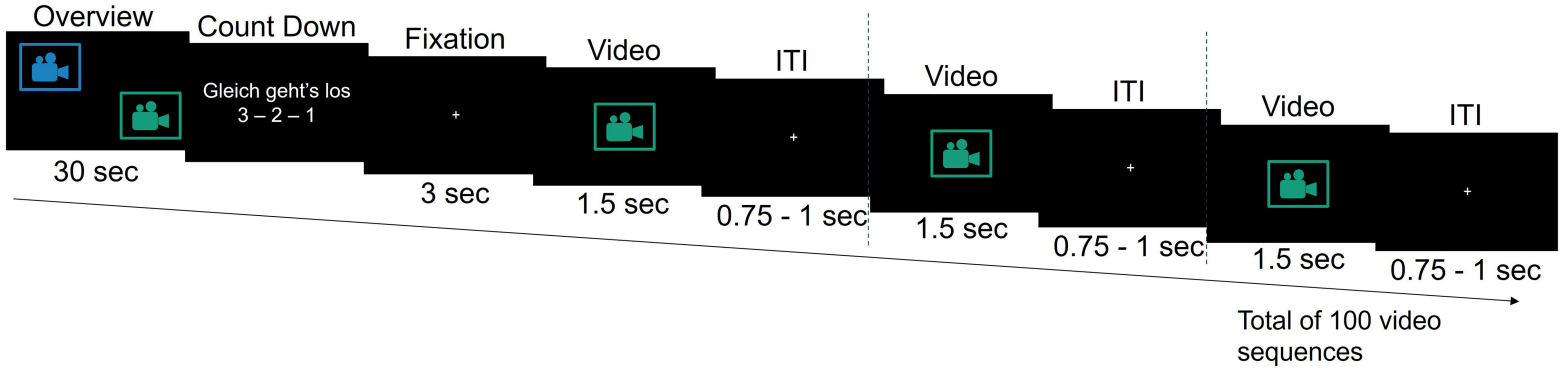
Overview of an experimental block in the robot action evaluation task. The overview phase at the beginning of each block includes two camera views. The following countdown is displayed in German (English: “It's about to start 3 - 2 - 1”). ITI, interstimulus interval.

Following a brief practice session consisting of 15 video sequences to familiarize them with the task and video material at the beginning of the experiment, participants were presented with 1,000 video sequences across 10 blocks. Each block included an overview video, a countdown, and randomized combination of 65 sequences showing optimal robotic actions and 35 showing suboptimal actions (100 sequences per block; Figures 1, 2). A 1-min break followed each block. The overview video at the beginning of each block depicted a sequence of optimal and suboptimal robotic actions from two viewpoints (zoomed-out in the top left corner of the screen and zoomed-in in the bottom right corner of the screen), providing context for the medical scenario and upcoming 100 zoomed-in video sequences. Each zoomed-in video sequence contained a single robotic action. It lasted 1.5 s and was followed by a jittered interstimulus interval ranging from 0.75 to 1 s, during which a fixation cross appeared at the center of the screen (Figure 2).

### 2.3 Data acquisition and preprocessing

EEG potentials were recorded according to the international 10–20 system with 64 electrodes and at a sampling rate of 1,000 Hz (actiCAP and BrainAmp, BrainProducts GmbH, Germany). The locations of the electrodes were Fp1, Fp2, Fz, AF3, AF4, AF7, AF8, F1, F2, F3, F4, F5, F6, F7, F8, FC1, FC2, FC3, FC4, FC5, FC6, FT7, FT8, FT9, FT10, Cz, C1, C2, C3, C4, C5, C6, T7, T8, CPz, CP1, CP2, CP3, CP4, CP5, CP6, TP7, TP8, TP9, TP10, Pz, P1, P2, P3, P4, P5, P6, P7, P8, POz, PO3, PO4, PO7, PO8, Oz, O1, O2, and Iz. The ground electrode was positioned on FPz and the reference electrode on FCz. Impedance of electrodes was kept below 25 *kΩ* at the beginning of the experiment.

All analyses were performed in Python and MNE Python (Gramfort et al., 2014). The EEG signals were de-trended and bandpass filtered using a fourth-order infinite impulse response (IIR) Butterworth filter with cut-off frequencies of 0.2 and 10 Hz (see also Iturrate et al., 2010, 2015; Kim et al., 2017, 2020; Vukelić et al., 2023). The signals were then segmented into 2.2-second epochs, each beginning 200 ms before the onset of each zoomed-in video sequence. Epoched data was subsequently down-sampled to 250 Hz. To remove cardiac, muscle, and ocular artifacts, epochs were cleaned using an independent component analysis (ICA; Chaumon et al., 2015; Hipp and Siegel, 2013; Lee et al., 1999) within an automated pipeline called FASTER (Nolan et al., 2010) as implemented in mne-python version 1.6.1 (Gramfort et al., 2014). To generate an electro-oculography (EOG) surrogate for the ICA, a virtual EOG channel was constructed using the frontal Fp1 and Fp2 electrode signals. After cleaning the signals, the epochs were baseline corrected by subtracting the mean amplitude of the time interval before the video onset (200 ms) and bad channels were interpolated per epoch using a spline interpolation (Gramfort et al., 2014; Nolan et al., 2010). Finally, the reference-free current source density (CSD) transformation was applied to the data to enhance spatial resolution by minimizing volume conduction effects and estimating local electrical activity (current sources and sinks) at the scalp surface (Perrin et al., 1989; Kayser and Tenke, 2015).

CSD is a mathematical transformation of EEG signals that estimates local current sources and sinks across the cortical surface at the sensor level. By computing the second spatial derivative of the electric potential field, it determines the spatial distribution and direction of current flow. Notably, the number of output channels matches the input channels, as the transformation is applied directly to the data from each electrode without changing the input dimensionality. CSD distinguishes between current sources (positive polarity) and sinks (negative polarity). In a CSD map, a source indicates outward current flow from a cortical region, reflecting reduced excitatory postsynaptic potentials (EPSPs). In contrast, a sink represents inward current flow linked to increased EPSPs (Perrin et al., 1989; Kayser and Tenke, 2015). This approach offers a more localized and directly interpretable representation of neural activity than standard reference-dependent EEG potentials (Perrin et al., 1989; Kayser and Tenke, 2015).

For subsequent analyses, the number of epochs was equalized across conditions by minimizing timing discrepancies across trial lists, ensuring an identical epoch count per condition.

### 2.4 Mass-univariate permutation-based clustering

To examine differences in brain signatures evoked by the robot actions, we used mass-univariate permutation-based spatiotemporal clustering (Maris and Oostenveld, 2007) with a paired *t*-test. The clustering was performed on contrast data, calculated by subject-wise subtracting suboptimal from optimal evoked responses.

Compared to traditional univariate approaches, such as performing an ANOVA or *t*-test on the mean or peak amplitude within a predefined time interval, mass-univariate statistics allow statistical testing at every location and time point (e.g., Maris and Oostenveld, 2007; Pernet et al., 2015; Groppe et al., 2011).

This approach is particularly advantageous when addressing variability in ERP latencies arising from experimental parameters, such as complex stimulus material (e.g., in the case of the P300; Bentin et al., 1999). However, the multiple comparisons problem-occurring when testing across many locations and time points-must be accounted for. Mass-univariate permutation-based spatiotemporal clustering addresses this issue by identifying clusters of contiguous samples (i.e., time points and sensors) that exhibit similar effects, thereby reducing the number of comparisons to the cluster level (Maris and Oostenveld, 2007). Neighboring effects (test statistics of time points and sensors) that exceed a predefined univariate cluster-forming threshold (here α < 0.05) are grouped into clusters. Statistical values (e.g., *t*- or *F*-values) within these clusters are aggregated, for instance by summing them, into cluster-mass scores (Maris and Oostenveld, 2007). Statistical significance is then determined by comparing the observed cluster-mass scores to a reference null distribution, generated via random resampling of condition labels (e.g., using Monte Carlo permutations or bootstrapping). A *p*-value is calculated for each cluster as the proportion of permutations in which the cluster-level statistic from the null distribution equals or exceeds the observed cluster-mass score obtained from the original dataset. To control the overall Type I error rate (false positives) across all clusters, only clusters with a *p*-value below a predefined group-level threshold (here α < 0.05) are considered statistically significant.

### 2.5 Temporal decoding with a linear machine learning model

Temporal decoding with subject-wise multivariate pattern analysis (MVPA) provides an alternative to mass-univariate analyses, offering enhanced sensitivity and statistical power (Holdgraf et al., 2017; Kriegeskorte and Douglas, 2019). MVPA leverages the multidimensional characteristics of neurophysiological data from each subject, thereby accounting for anatomical and functional inter-individual neural variability (Marsicano et al., 2024).

**Figure 3:**
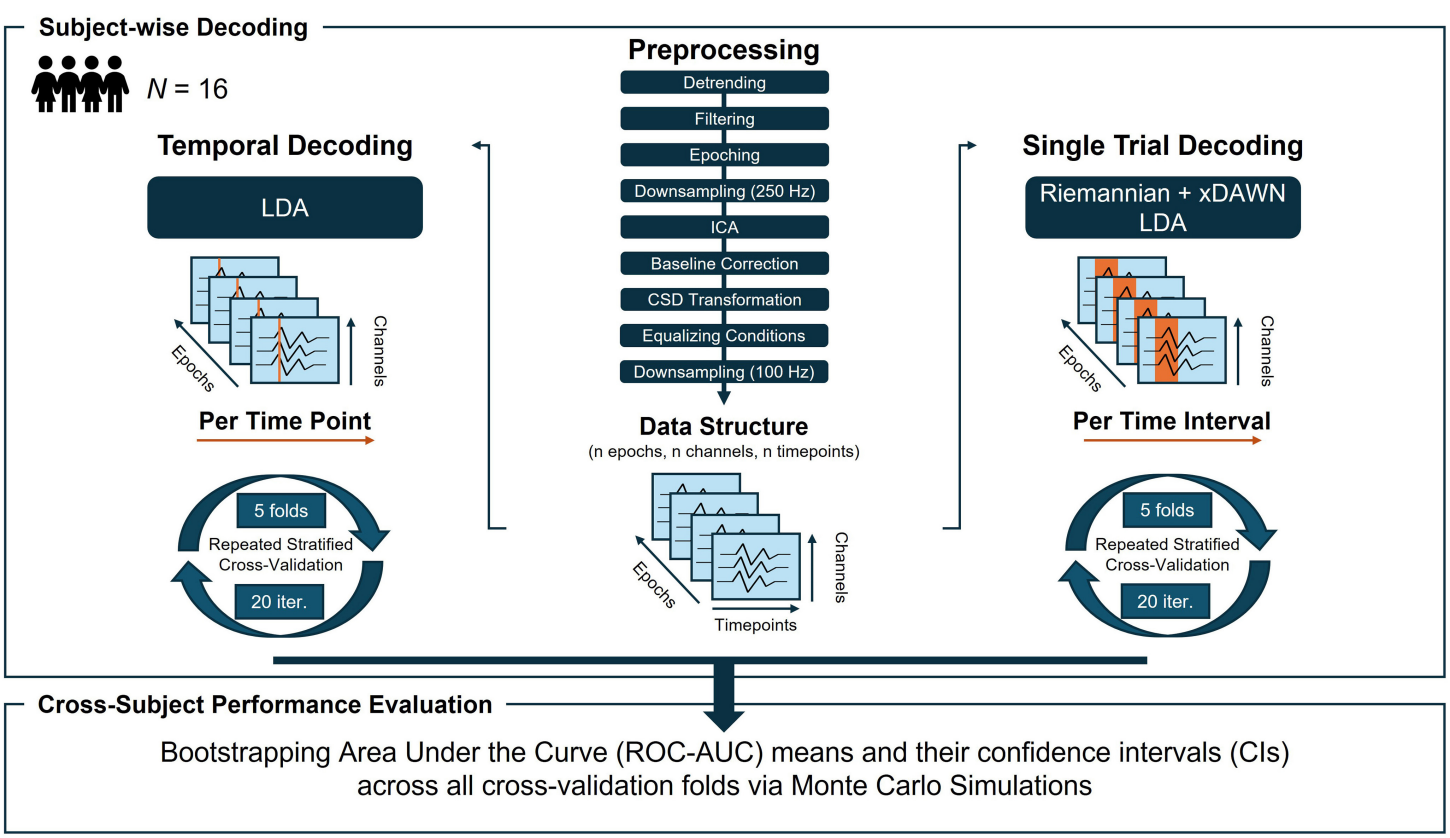
Overview of the preprocessing steps, data structure and machine learning pipeline for the **(left column)** temporal decoding and **(right column)** single-trial decoding. *N*, sample size; LDA, linear discriminant analysis; Iter, iterations.

Figure 3 illustrates the input data structure and pipeline steps applied in temporal decoding. For the machine-learning (ML) based analyses, epoched data were downsampled to 100 Hz to reduce computational costs. Linear discriminant analysis (LDA), using a least-squares solution and automatic shrinkage via the Ledoit-Wolf lemma (as implemented in scikit-learn version 1.4.1), was applied as a sliding supervised ML algorithm (i.e., the Base Estimator) on a time-point-by-time-point basis (implemented in mne-python version 1.6.1; Gramfort et al., 2014). The data of each participant (shape: n epochs, n channels, n timepoints; Figure 3) was split into training and testing sets using a repeated stratified five-fold cross-validation with 20 iterations, resulting in 100 folds per time point. In total, 220 (timepoints) × 100 (cross-validation folds) × 16 (participants) models were trained and fitted in the time decoding. The Area Under the Receiver Operating Characteristic Curve (ROC-AUC, henceforth referred to as AUC) was used as performance metric. Classification performance was statistically evaluated by bootstrapping the AUC scores across participants and folds in a Monte Carlo simulation (MCS; 5,000 iterations), yielding the bootstrapped mean and its 95% confidence interval (CI; Cumming, 2014). Time intervals were considered significant if the lower CI boundary of the average LDA performance exceeded the upper CI boundary of an average dummy performance (i.e., an empirical baseline estimated by chance-level stratified classification in scikit-learn version 1.4.1).

After fitting the linear models, model decoding weights were transformed into activation patterns representing their contribution to classification through inverse computations (Haufe et al., 2014). These activation patterns were averaged across participants and visualized using topographic maps. A spatiotemporal mask was applied to identify statistically significant activation patterns using univariate bootstrapped means and CIs (MCS with 5,000 iterations). Only patterns at electrode positions where the CI for the average evoked response contrast (suboptimal–optimal robot actions) excluded zero were considered significant and visualized. Positive values in the activation patterns indicate that the region contributes to the classification of evaluated suboptimal robot actions, whereas negative values indicate a contribution to the classification of evaluated optimal robot actions. Pattern values closer to zero indicate lower confidence in their contribution. To assess the relationship between evoked response amplitudes and significant patterns, the time course of contributing regions was visualized, along with bootstrapped means and CIs for each condition at the time point of maximal classification performance.

### 2.6 Single-trial decoding

In the final analysis, we decoded the observer's evaluation of robot actions from brain signatures on a trial-by-trial basis using three distinct time intervals for feature extraction identified through MVPA time decoding (see Figure 3 for an illustrative overview). These intervals were defined as (1) 0–750 ms, (2) 750–1,350 ms, and (3) 1,350–2,000 ms after video onset. As in the time decoding, data were downsampled to 100 Hz to reduce computational costs.

An LDA classifier with automatically extracted features based on Riemannian geometry has been proven effective for state decoding in passive BCIs (Lotte et al., 2018; Vukelić et al., 2023) and was, thus, applied to each time interval in a within-subject single-trial decoding (implemented in pyRiemann; version 0.5). The Riemannian-based method operates directly on the epoched EEG time series (data shape: n epochs, n channels, n timepoints; Figure 3), obviating the need for manual feature extraction. It converts the EEG time series into symmetric positive definite (SPD) covariance matrices and applies Riemannian geometry to analyse these matrices (Congedo et al., 2017; Appriou et al., 2020; Vukelić et al., 2023). In the Riemannian manifold, covariance matrices were spatially filtered with the xDAWN algorithm (Rivet et al., 2009) before being projected into tangent space for transformation into Euclidean vectors (Barachant et al., 2011). This tangent space projection preserves the manifold structure while enabling effective classification (Appriou et al., 2020).

Classification was performed using an LDA classifier (with default settings as implemented in scikit-learn version 1.4.1). Performance was quantified using a repeated stratified k-fold cross-validation (five splits, 20 iterations) with AUC as metric. As with temporal decoding, a dummy classifier estimated chance-level performance. Non-parametric bootstrapping of classification scores across folds and subjects yielded the average performance and corresponding CI for each classifier, enabling statistical evaluation (Cumming, 2014).

## 3 Results

### 3.1 Mass-univariate permutation-based clustering

The non-parametric permutation-based clustering identified significant spatiotemporal differences in evoked responses when observing suboptimal compared to optimal robot actions across five clusters.

**Figure 4:**
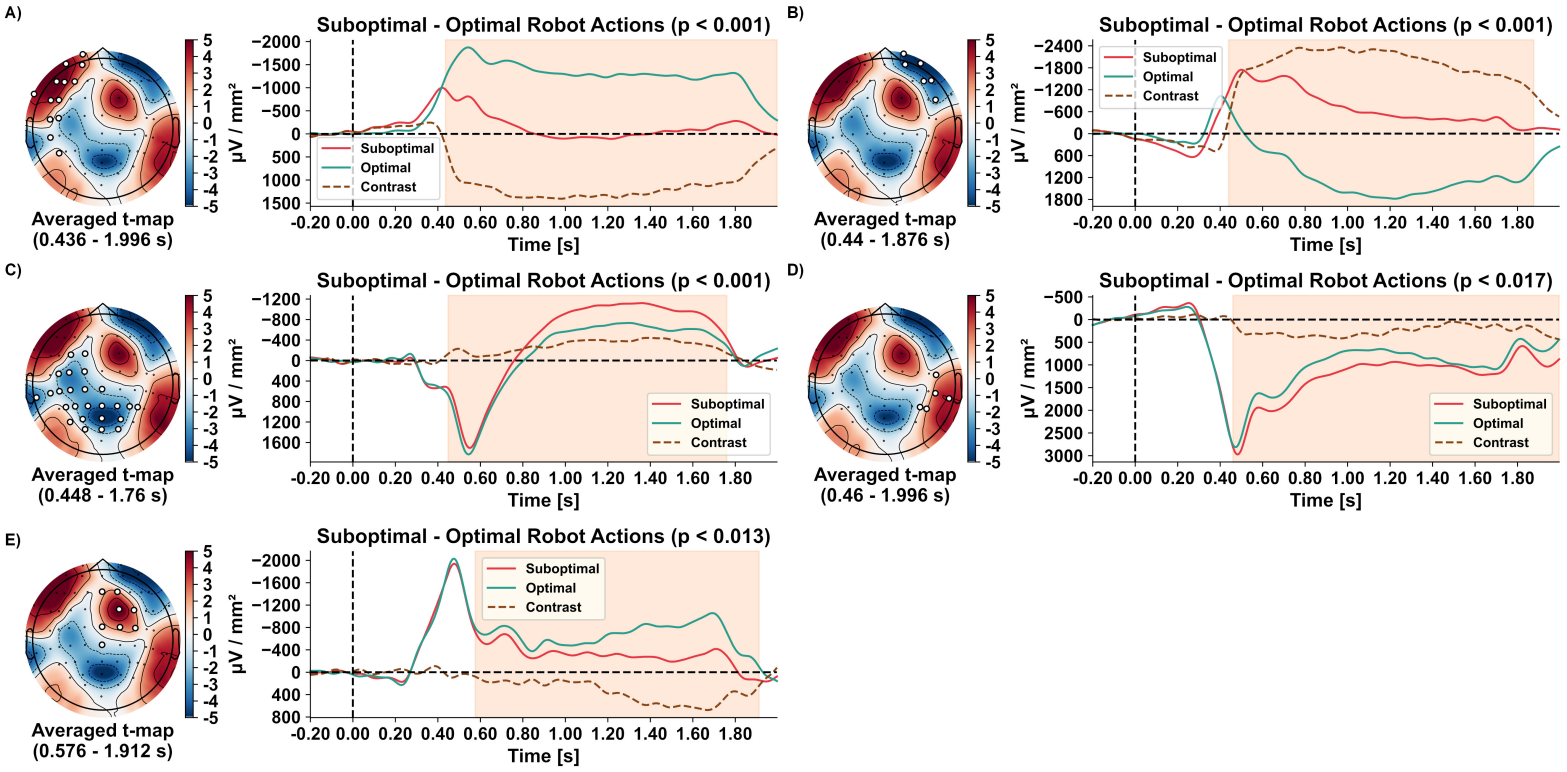
Spatio-temporal clusters **(A–E)** with topographical maps of averaged *t*-values, along with evoked responses for each condition and their contrast. Significant electrode positions for each cluster are indicated by filled white circles. Grand averages (*n* = 16) of the evoked responses during observation of optimal (green) and suboptimal (red) robot actions are shown over time, including their contrasts (suboptimal–optimal; brown dashed line). The time ranges of significant clusters are highlighted in orange.

The first two clusters emerged ~440 ms after video onset, revealing lateralised frontal responses. Observing suboptimal robot actions resulted in a reduced left-hemispheric frontal current sink (Figure 4A; 13 electrodes; *p* < 0.001) and an enhanced right-hemispheric frontal current sink (Figure 4B; six electrodes; *p* < 0.001). In electrodes overlying right-hemispheric frontal regions, observing optimal robot actions was even associated with current sources (i.e., a positive deflection) from around 500 ms until the analysis window's end (Figure 4B). The third cluster, including 25 electrodes over occipital, parietal, and left temporal regions, emerged at 448 ms. It differentiated robot actions by showing a reduced current source peak around 550 ms, followed by an increased current sink from 800 to 1,760 ms for suboptimal compared to optimal actions (Figure 4C; *p < 0.001*). The fourth cluster, with five electrodes over right parieto-temporal regions, appeared at 460 ms, showing increased current sources for suboptimal actions (Figure 4D; *p* < 0.017). Finally, the fifth cluster over fronto-central regions, emerging after 576 ms, showed a decreased current sink for suboptimal actions (Figure 4E; eight electrodes; *p* < 0.013). All clusters persisted almost until the end of the 2-s analysis interval (1,760–1,996 ms).

### 3.2 Temporal decoding with a linear machine learning model

Temporal decoding using MVPA and LDA successfully distinguished the brain signatures evoked by observing optimal vs. suboptimal robot actions.

**Figure 5:**
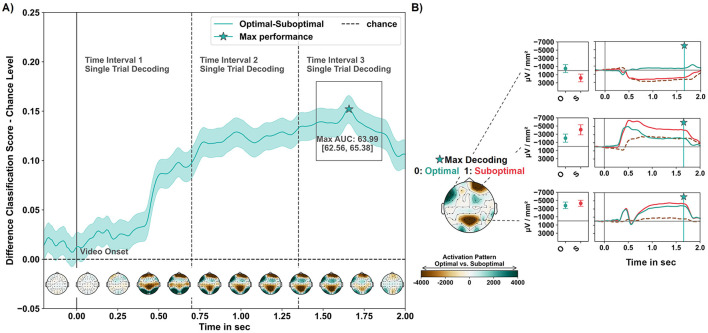
Classification performance in the MVPA temporal decoding with LDA. **(A)** Average LDA temporal classification performance, including the corresponding CI band across folds and subjects, is presented relative to the estimated chance level (upper CI boundary of the average dummy classifier performance). Below, the average activation patterns derived from model weight coefficients are depicted. Patterns were spatio-temporally masked using bootstrapped CIs and averaged over time intervals of 200 ms starting 200 ms before to 2,000 ms after the onset of the video. The star icon indicates the peak (max at 1,658 ms) of above-chance level classification performance. **(B)** Activation pattern of the time point of peak decoding performance, along with the evoked responses per condition in the regions of meaningful contribution at the maximum decoding time point and as time series along the analysis interval (dashed gray line: contrast suboptimal–optimal). Positive pattern values are associated with classifying observed optimal robot actions, while negative values in activation patterns are associated with observed suboptimal robot actions.

The empirical chance level of the dummy classifier was estimated at an AUC score of 48.4 95% CI [48.06, 48.79]. In later intervals, beginning 750 ms post-stimulus onset and continuing until the end of the 2-s analysis period, classification performance consistently exceeded a 60% AUC score. The classification performance varied over the analysis interval, with a standard deviation of 4.13 (4.11, 4.13). The highest classification performance was observed after 1,658 ms with an AUC score of 63.99 95% CI [62.56, 65.38], representing a difference of 15.21 (95% CI [13.78, 16.6]) to the upper CI boundary of the mean chance performance (see Figure 5A).

At the peak decoding time, significant activation patterns highlighted three regions of interest that differentiated between optimal and suboptimal robot actions. These regions included electrodes over the right frontal, left fronto-temporal, and mid-parietal areas, corresponding to three clusters identified in the mass-univariate permutation-based analysis.

Figure 5B shows the relationship between classification-contributing regions and the brain signatures evoked in these regions by the conditions. The pattern that classified suboptimal robot actions comprised electrodes positioned over a left fronto-temporal region (F7 and FT9) and revealed a current source for suboptimal actions, while optimal robot actions elicited a current sink. Two other regions contributed to classifying optimal robot actions: Current sinks in a right frontal electrode (Fp2) and electrodes overlying the mid-parietal region (P1, Pz, P2) were reduced for evaluating optimal compared to suboptimal actions.

### 3.3 Single-trial decoding

**Figure 6:**
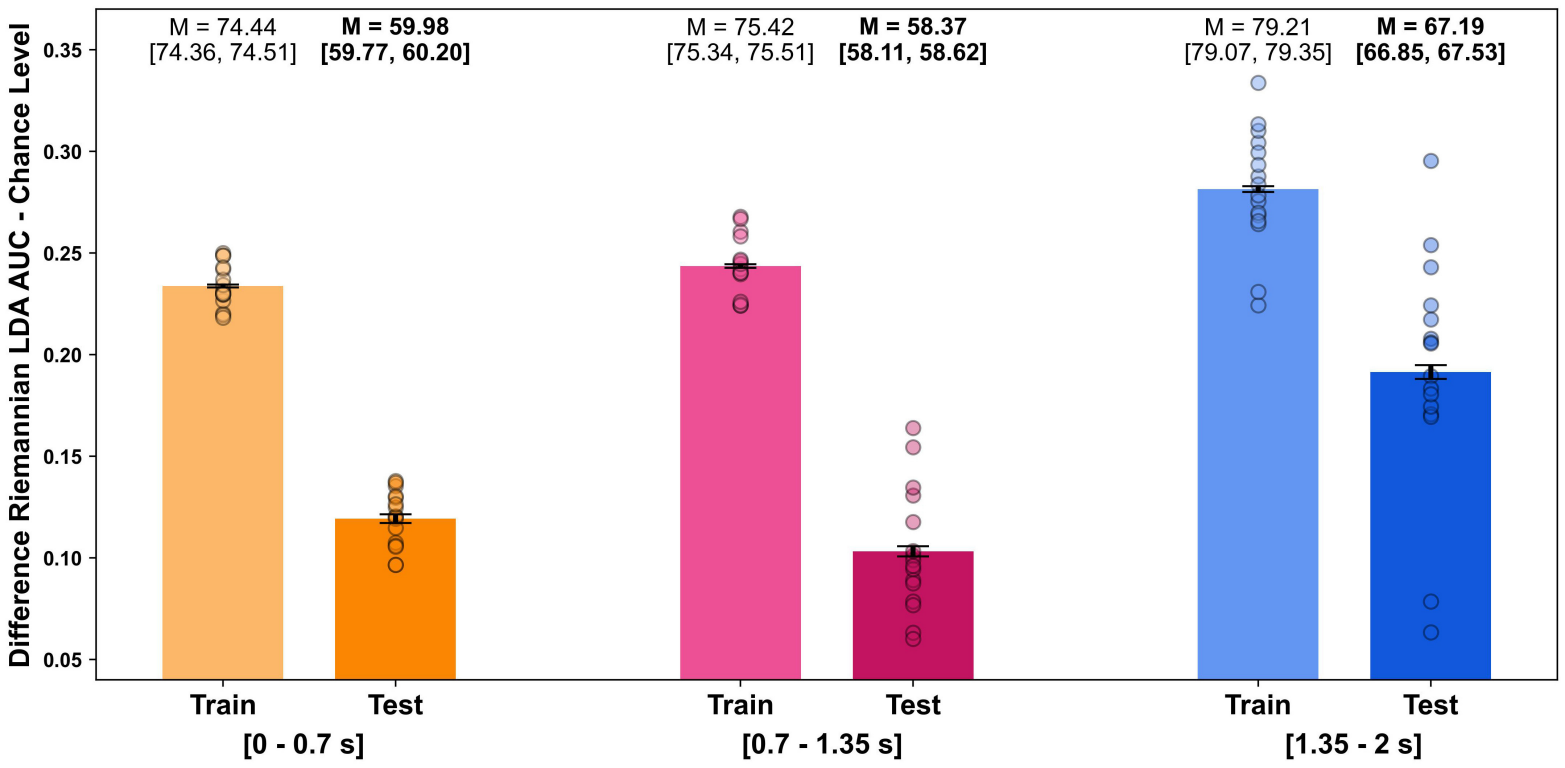
Riemannian LDA single-trial decoding performance by time interval. Bar plots display the average AUC classification scores of the Riemannian LDA relative to the upper CI boundary of the estimated chance level (dummy performance: train AUC = 48.02, 95% CI [47.99, 51.06]; test AUC = 51.04, 95% CI [51.02, 51.06]). The mean AUC score (M) and its 95% CI ([]), estimated via bootstrapping and represented by error bars, are displayed above each bar (for training and test datasets across time intervals). Individual subject decoding performances are depicted as scattered dots. Time intervals of the decoding were (1) 0 to 700 ms, (2) 700 to 1,350 ms, and (3) 1,350 to 2,000 ms after video onset.

In the single-trial decoding of robot performance evaluations, the Riemannian LDA combined with xDawn spatial filtering yielded classification results above chance level for all selected time intervals (dummy performance: train AUC = 48.21, 95% CI [48.15, 48.28]; test AUC = 51.37, 95% CI [51.36, 51.38]). The highest classification performance was observed using the latest interval including evoked responses from 1,350 to 2,000 ms after video onset, with a test AUC of 67.19 (95% CI [66.85, 67.53]). This interval also included the time point of peak decoding performance in the MVPA-based temporal decoding. In contrast, earlier intervals cropped before 750 ms post-stimulus showed a significant decrease in performance, with test AUCs of 59.98 (95% CI [59.77, 60.20]) for an interval from 0 to 700 ms and 58.37 (95% CI [58.11, 58.62]) for an interval from 700 to 1,350 ms (Figure 6).

## 4 Discussion

Our study identified distinct spatiotemporal brain dynamics that reliably differentiate the mental performance evaluation of optimal and suboptimal robotic actions observed in video excerpts of laparoscopic training procedures.

We assessed the robustness of neural signatures by employing surface Laplacian transformations to enhance the spatial resolution of evoked responses (see Somon et al., 2019) and two complementary data-driven methods - a mass-univariate permutation-based clustering and multivariate pattern analysis (MVPA) temporal decoding. The identified discriminative spatiotemporal brain signatures suggest that differentiation between optimal and suboptimal actions does not occur during early perceptual stages but rather at later evaluative stages (Somon et al., 2017; Ferrez and Millán, 2005; Chavarriaga et al., 2010; Oliveira et al., 2007). This finding was observed despite a perceptual component introduced by LED feedback in the evaluation task.

In addition to analyzing stimulus-locked evoked responses, we examined single-trial decoding performance of robot action evaluations across different time intervals of averaged evoked brain responses. The Riemannian LDA with xDawn filtering reliably classified observers' electrophysiological responses to optimal and suboptimal robot actions on a trial-by-trial level. Temporal dynamics of classification performance revealed that late intervals (from 1,350 to 2,000 ms post-stimulus) significantly outperformed earlier intervals aligning with findings from MVPA temporal decoding. This indicates that the most informative brain patterns are linked to attentional and evaluative processes related to prediction violations and unexpected events (Somon et al., 2017; Chavarriaga et al., 2010; Ferrez and Millán, 2005; Oliveira et al., 2007).

### 4.1 Convergent findings for evoked responses differentiating robot performance

Our clustering analyses revealed five spatiotemporal brain signatures associated with robot performance evaluation, of which three were replicated in the temporal decoding. The other two clusters including electrodes localized over right mid-fronto-central and temporal areas were exclusively identified in the mass-univariate analysis. Thus, they exhibited limited reliability as distinctive patterns for evaluating robot performance in near-naturalistic scenarios. Therefore, the next section focuses on the remaining three signatures located over the left fronto-temporal, right frontal, and mid-occipito-parietal regions.

#### 4.1.1 Left fronto-temporal spatiotemporal signature

Evoked responses in electrodes overlying left fronto-temporal regions differentiated the evaluation of optimal and suboptimal robot performance in both the clustering (Figure 4A) and temporal decoding (Figure 5B, upper row). This brain dynamic is characterized by differences in current direction-switching around 420 ms post-stimulus onset between suboptimal and optimal robot actions. Observing suboptimal performance evoked a persistent current source (see Figure 5B, upper row), while optimal performance elicited a sustained current sink during late time intervals (see Figures 4A, 5B, upper row).

The late shift to a current source during suboptimal actions may indicate the allocation of additional cognitive resources for conflict processing and deviation detection (Botvinick et al., 2001; Ullsperger et al., 2014; Bartholow et al., 2005; Pailing and Segalowitz, 2004). Although typical oERN or oPE responses were not observed in this study, the sustained fronto-temporal responses align with components such as the P300, late positive potential (LPP) and P600 (Somon et al., 2017; Sassenhagen et al., 2014; Oliveira et al., 2007). The P300 and LPP are positive deflections that typically emerge around 300 ms after significant and emotionally salient stimuli, respectively, at centroparietal electrode sites (Polich, 2007; see Hajcak and Foti, 2020 for review). The P300 appears as a broad peak, while the LPP can be sustained for up to 1,000 ms or more. Their amplitudes increase in response to motivationally significant but also deviant and uncertain stimuli (Scheffers and Coles, 2000; Sutton et al., 1965), indicating sustained attentional allocation toward these stimuli (Ridderinkhof et al., 2009; Hajcak and Foti, 2020; Falkenstein et al., 2000). The P600, initially linked to processing linguistic anomalies (Sassenhagen et al., 2014), has also been observed during error processing in choice-reaction time tasks with enhanced amplitudes following errors Falkenstein et al. (1991).

In summary, the sustained current source observed during suboptimal robot actions likely reflects increased cognitive and attentional engagement in a persistent evaluative stance. This state likely facilitates conflict detection by assessing action accuracy and adequacy, monitors deviations, and supports cognitive flexibility.

#### 4.1.2 Right frontal and mid-occipito-parietal spatiotemporal signatures

In addition to the left fronto-temporal signature, we observed two spatiotemporal signatures characterized by enhanced current sinks for evaluated suboptimal robot performance in both, the cluster analysis (Figures 4B, C) and temporal decoding activation patterns (Figure 5B, middle and lower row).

After ~300 ms, a right frontal current sink emerged, peaking between 400 and 600 ms, with a delayed but pronounced deflection in response to suboptimal actions (Figures 4B, 5B, middle). Another spatiotemporal brain signature, indicative of suboptimal actions and located over mid-occipito-parietal areas, appeared between 350 and ~500 ms (Figures 4C, 5B, lower row). This mid-occipito-parietal signature is characterized by a current sink deflection in response to both optimal and suboptimal robot actions, followed by a short time interval of current source with a peak at 550 ms. Afterwards, another directional switch from source to sink is observed, occurring around 600 ms second in the decoding and 700 ms in the clustering analysis. In both analyses, this sustained current sink in late time intervals after stimulus onset was more pronounced when observing suboptimal compared to optimal robot performance.

These time windows and sustained current sinks for suboptimal robot actions likely reflect a combination of a delayed oERN and an interaction ERN/N400 (Chavarriaga et al., 2010; Ferrez and Millán, 2005; Ferrez and Millán, 2008; Somon et al., 2017). The delay in evoked response intervals is potentially attributable to the erroneous robot action occurring shortly after the video onset. Notably, the N400 has previously been observed in non-linguistic contexts over parietal areas in response to unexpected motor sequences (Balconi and Vitaloni, 2014). Both ERP components are amplified when observing erroneous, suboptimal actions. In their sustained form, they may reflect ongoing quality evaluation, signaling deviations from predicted trajectories and expected movements, thereby indicating suboptimal performance.

To summarize, through temporal decoding and clustering analyses, we identified three consistent spatiotemporal signatures that distinguish the evaluation of optimal and suboptimal robot performance. A left fronto-temporal signature, characterized by an enhanced current source resembling ERP components such as the P300, LPP, and P600, suggests increased attentional allocation and sustained evaluation of suboptimal robot actions. Furthermore, right frontal and mid-occipito-parietal signatures displayed amplified current sinks in response to suboptimal actions, suggesting prediction-based processing of deviations and errors, consistent with the oERN and interaction-based ERN/N400.

### 4.2 Effects of task load and video stimulus material

The identified discriminative evoked signatures reflect a sustained, step-by-step evaluation of robot actions from continuous video excerpts. They persisted even after deviations from expected (optimal) performance were detected. Consequently, optimal robot actions were characterized by the absence of deviations throughout the entire video. In our specific task, participants were required to monitor and mentally assess multiple aspects of the action, including the position, length, and pressure of the intended cut. Thus, even if the robot correctly reached the target position, participants needed to verify that all criteria were met. Accordingly, it is noteworthy that the continuous video stimulus, coupled with the ongoing monitoring and evaluation of robot actions in an applied scenario, likely imposed a substantial perceptual and cognitive load on participants.

This task-induced load may have reduced differences in the amplitude of evoked responses between observed suboptimal and optimal actions (Somon et al., 2017, 2019; see Endrass et al., 2012a,b for load effects during self-monitoring). It could explain the lack of modulated amplitudes in early components during the observation of suboptimal actions. In addition, although the task instructions aimed to emphasize the importance of errors and the potentially serious consequences of mistakes in laparoscopic surgery, the absence of a modulated Pe component in response to suboptimal robot actions may be due to the low (self-related) relevance of negative outcomes for participants in a passive observation role (Chavarriaga et al., 2010; Somon et al., 2017).

To conclude, given that task-induced cognitive load on the observer may be inherently present and unavoidable in real-world applications, further investigation is warranted to ensure ecologically valid and robust correlates of performance assessment.

### 4.3 Limitations and future directions

The study offers valuable insights into the neural mechanisms underlying robot performance evaluation and error monitoring in a near-naturalistic laparoscopic surgical training context. However, several limitations must be considered.

To aid non-medical participants in judging whether the robot's actions were optimal or suboptimal - particularly for subtle criteria such as applied pressure - LEDs were placed along the tissue to be cut. While this LED feedback during suboptimal actions (i.e., LEDs remained lit) vs. optimal actions (LEDs turned off) was essential for participants' understanding and engagement, it may have influenced evoked responses, introducing a perceptual component to the task and complicating comparisons with previous studies. Future research on passive BCIs for robotic training should explore alternative guidance methods and additional information sources, such as haptic feedback representing applied pressure, to reduce reliance on perceptual feedback and better isolate evaluative processes.

Our findings revealed a lateralised error-related brain signature, marked by enhanced left fronto-temporal current sources and right frontal current sinks. This lateralisation has not been previously reported and may represent a novel correlate of performance assessment during robot action monitoring in applied scenarios. Given the rather small sample size in this study, further research should confirm the robustness and replicability of the identified spatiotemporal brain signatures linked to robot action evaluation in realistic scenarios.

Moreover, including medical students or even physicians would enable an assessment of the impact of expert knowledge. Future research should also explore the potential effects of participant fatigue or fluctuating task engagement throughout prolonged video sequences, as this may introduce variability in evoked responses. Addressing these factors could further clarify the robustness of our identified brain signatures.

Precise onset detection of suboptimal actions is challenging in near-naturalistic experiments and is often shaped by subjective observer criteria. Therefore, eye-related measures, such as fixations (Simola et al., 2015; Ladouce et al., 2022) or blinks (Alyan et al., 2023), may provide an ecologically valid approach to further investigate attentional shifts toward significant deviations and the associated evaluative processing.

Combining deviation onset detection through eye-based approaches with findings from temporal and single-trial decoding establishes a foundation for developing passive BCIs to reliably label robot actions for reinforcement-learning-based training paradigms. In our study, the most informative signals were extracted from late evoked responses linked to attentional or evaluative processes. Consequently, BCI algorithms should focus on these late evaluative intervals (beyond 1,000 ms after eye-based deviation detection) to enhance decoding accuracy. However, it is important to note that while late evoked responses are suitable for training robots, the delay of a few hundred milliseconds following error detection may restrict their effectiveness for real-time interventions. Such real-time interventions could provide a safeguard in robot-assisted surgeries. To overcome this limitation, future studies could investigate a multisensory decoding approach that integrates electrophysiological, peripheral-physiological, and eye-based data, combined with a conservative stop criterion (high sensitivity/true positive rate), to develop a system capable of intervening and eliminating suboptimal robotic actions in real-life surgical scenarios.

The next steps toward BCI-assisted robot training in real-world settings include replicating these findings in (a) dual-task paradigms that simulate collaborative scenarios with individual and shared tasks, and (b) using mobile, dry EEG systems suited for unobtrusive, everyday measurements (e.g., Vukelić et al., 2023).

## 5 Conclusion

Our study reveals three robust spatiotemporal brain signatures that distinguish between evaluated optimal and suboptimal robotic actions during laparoscopic training. The findings emphasize the critical role of late-stage evaluative brain processes in detecting deviations in robotic performance. Specifically, the left fronto-temporal signature, associated with ERP components such as the P300, LPP, and P600, indicates sustained attentional and evaluative engagement in response to suboptimal actions. Additionally, amplified current sinks in right frontal and mid-occipito-parietal regions, consistent with error-related responses like the oERN and ERN/N400, suggest prediction-based processing of errors and deviations.

By delineating distinct electrophysiological patterns, our results deepen the understanding of the neural mechanisms underpinning mental assessments of robotic performance in near-naturalistic scenarios. These insights hold promise for advancing passive BCIs capable of facilitating real-time, automated evaluations in robotic training and collaborative surgical contexts. The research highlights the role of late-stage electrophysiological responses, linked to attentional and evaluative processes, in detecting significant deviations from optimal robotic actions. Integrating these findings into reinforcement-learning-based training frameworks could reduce reliance on explicit feedback from human instructors, enabling more efficient and intuitive human-centered robotic training systems.

## Data Availability

The datasets presented in this study can be found in an online OSF repository and are accessible upon request at https://osf.io/6ndsv/.
