# Supplementary material for: Evaluating robotic actions: spatiotemporal brain dynamics of performance assessment in robot-assisted laparoscopic training
Source: Front Neuroergon. 2025 Feb 19;6:1535799. doi: 10.3389/fnrgo.2025.1535799 (PMC11880255; doi:10.3389/fnrgo.2025.1535799)

Supplementary Material

1 STIMULUS MATERIAL

A Panda robotic arm (Franka Emika GmbH, Munich, Germany) was tele-navigated to produce video sequences of optimal and suboptimal robotic actions for simulated laparoscopic surgery (Supplementary Figure S1B). Participants, however, were informed that the robotic actions were fully autonomous. Tissue-cutting procedures were simulated using a rod on a phantom torso model with replicated organs.

A total of 354 video sequences were created for the experiment and presented in random order with repetitions after each complete cycle. These videos varied by camera setup, target organ (see Supplementary Figure S1C), and error type (Supplementary Table S1). In terms of camera positioning, the robotic rod was either minimally visible, positioned slightly to the left, or visible, positioned slightly to the right. The target organs included the right kidney, stomach, and spleen, each equipped with single LED point sensors, and the left kidney, which featured a line sensor comprising a row of seven LEDs (Figure 1A-D and Supplementary Figure S1A). The robot's task was to apply pressure to the marked tissue with point sensors, while with the line sensor, it was to move the rod along the organ's surface. The robot introduced two types of errors: a complete miss of the target organ (no contact; Figure 1B and D) or insufficient pressure or cut length upon contact (Figure 1C). The videos were varied primarily to prevent anticipatory judgments and to create a generalisable and realistic environment. However, only the classification of robot actions into optimal and suboptimal (i.e., failed) was considered in the analyses.

2 PILOT STUDY

We conducted a preliminary behavioural study with nine participants (four male, three female; age: $M = 24.33, SD = 2.00$, range = 21 - 28 years) to (a) confirm the comprehensibility of the stimulus material and (b) verify that participants could attain high classification accuracy. Results showed that participants correctly classified the robot actions in 96.25% of the observed videos, with a mean response time of 0.708 seconds. A linear mixed model was used to predict the condition (optimal vs. suboptimal robot action) based on response time, classification accuracy, and misses, with random intercepts for participants to account for inter-individual variability. The fixed effect of response time was significant, β = 13.52, 95% CI [5.20, 21.83], Z = 3.19, $p < .001$, indicating that longer response times were associated with the observation of suboptimal robot actions. In contrast, neither classification accuracy (β = 9.36 95% CI [-1.97), 20.70], Z = 1.62, $p = .105$) nor misses (β = 9.20, 95% CI [-2.30, 20.71], Z = 1.57, $p = .117$) were significant predictors of robot action condition.

3 EVOKED RESPONSES OVER TIME

Figure S2 presents topographic maps illustrating the spatial distribution of evoked responses over time for the two conditions, optimal (first row) and suboptimal (middle row) robotic actions, as well as their difference (suboptimal contrasted with optimal robotic actions). Each column corresponds to a different time window (averaged over 200 ms intervals), capturing distinct phases of evoked potentials from 200 ms before to 2,000 ms after video onset. The colour-coded distributions represent the amplitude of the evoked responses, with red and blue indicating positive (sources) and negative (sinks) currents, respectively, as calculated using the current source density (CSD) method. The figure reveals the temporal evolution of

evoked responses. It highlights regions of interest, including fronto-temporal, parietal, and occipital areas, which differentiate performance assessments while monitoring optimal and suboptimal robotic actions.

4 SUPPLEMENTARY FIGURES AND TABLES

Figure S1. Overview of the experimental setup for the robot-assisted medical training simulation. A) Excerpt from an exemplary zoomed-out overview video. B) Schematic of the technical setup, featuring the tele-manipulation of the robotic arm. C) Excerpt from an exemplary zoomed-out overview video depicting the setup, comprising a phantom torso with replicated organs and integrated LED sensors, alongside the robotic arm with an attached camera.

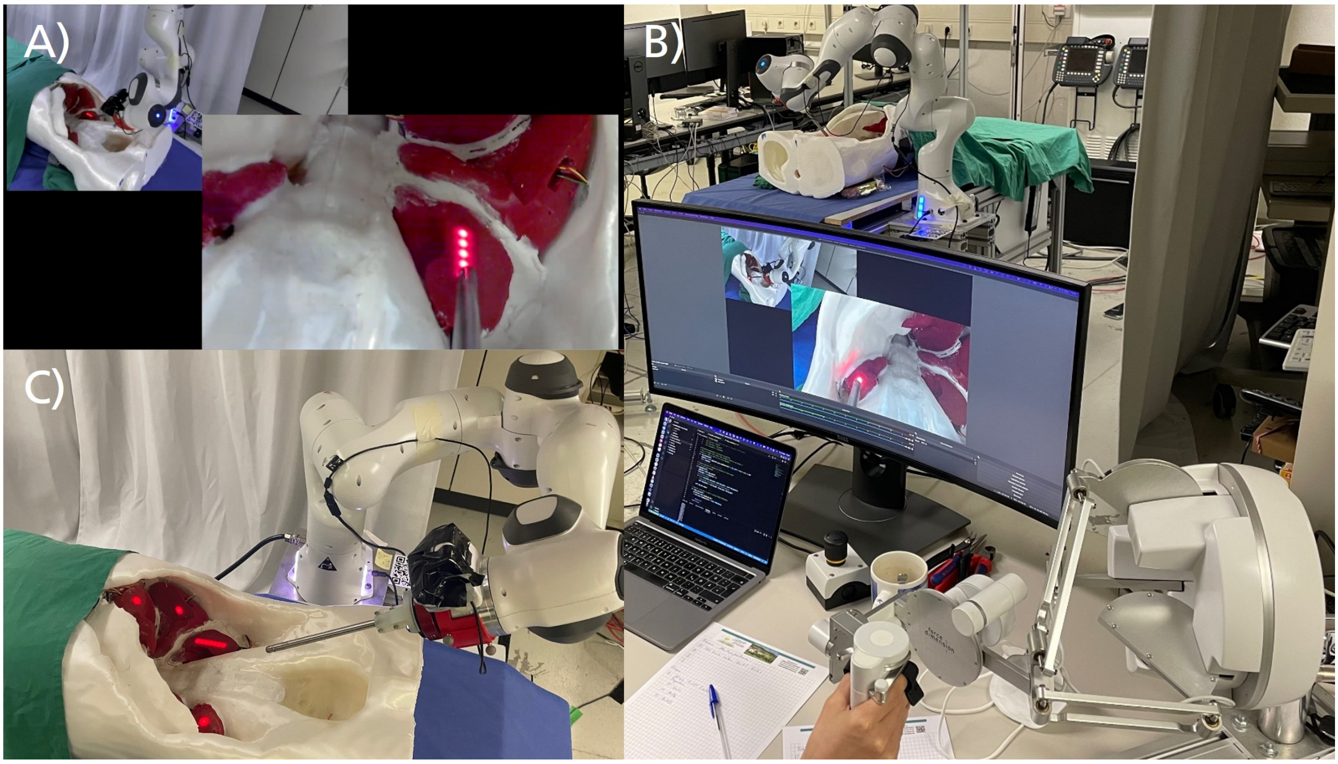

Table S1. Video sequence variations categorised based on the visible organ, type of error and two different camera setups (1: Rod is not very visible and slightly to the left in the picture, 2: Rod is visible and slightly to the right in the picture).

Target Organ	Camera Set-up	No Error	Missed	Not Completed
Right kidney with point sensor	1	14	3	2
	2	59	6	8
Stomach with point sensor	1	3	2	4
	2	46	11	6
Spleen with point sensor	1	21	1	2
	2	20	4	5
Left kidney with line sensor	1	28	11	11
	2	51	14	22

Figure S2. Topographic maps illustrating the spatial distribution of grand-average evoked responses (current sources and sinks computed using surface Laplacian current source density) over time for optimal (upper row) and suboptimal (middle row) robotic actions, as well as their contrast suboptimal-optimal robotic actions (bottom row). Evoked responses were averaged over time intervals of 200 ms starting 200 ms before to 2,000 ms after the onset of the video.

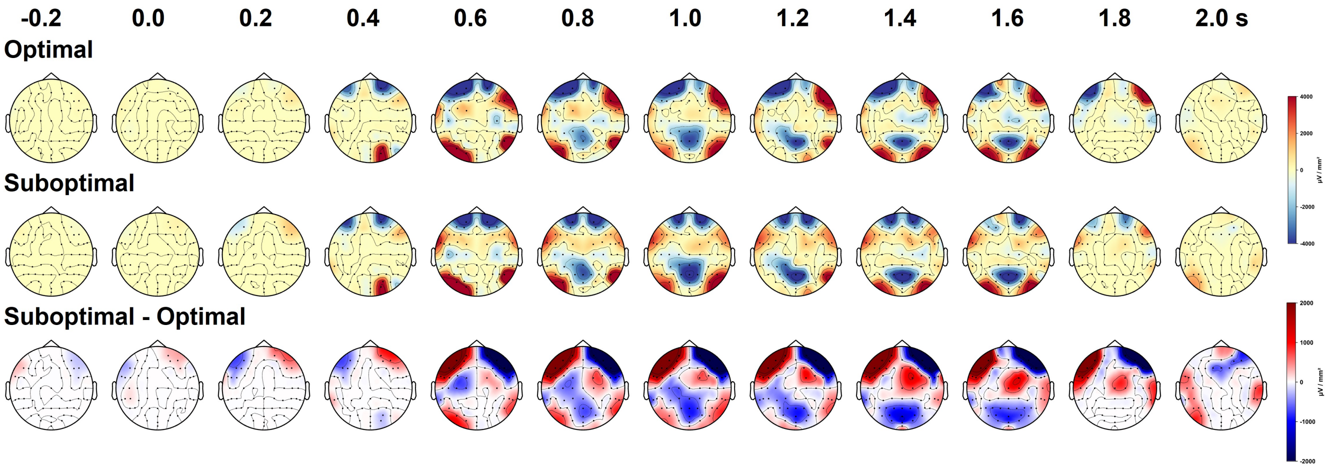

Supplement: Supplementary file 2 [file Data_Sheet_1.pdf]
